# Extracorporeal Membrane Oxygenation Uses in Refractory Cardiogenic Shock After Open-Heart Surgery

**DOI:** 10.21470/1678-9741-2022-0344

**Published:** 2023-10-23

**Authors:** Pablo Salazar Elizalde, German J. Chaud, Joaquín Gundelach, Barbara Gaete, Marcos Durand, Ignacio Cuadra, Sinthya Provoste, Enrique Yanten, Marcelo Tiznado, Cristóbal Alvarado

**Affiliations:** 1 Cardiac Surgery Department and ECMO Unit, Las Higueras Hospital, Talcahuano, Chile; 2 Biomedical Research Unit, Las Higueras Hospital, Talcahuano, Chile; 3 Basic Sciences Department, School of Medicine, Universidad Católica de la Santísima Concepción, Concepción, Chile

**Keywords:** Extracorporeal Membrane Oxygenation, Stroke Volume, Heart Failure, Left Ventricular Function, Morbidity, Catheterization

## Abstract

**Introduction:**

Extracorporeal membrane oxygenation (ECMO) is the first-line therapy for temporary mechanical circulatory support allowing cardiac and pulmonary recovery or as a bridge to further therapeutic alternatives. The aim of this study was to report clinical outcomes in adult patients with refractory cardiac failure after open-heart surgery undergoing ECMO in a single center with an ECMO unit in Chile.

**Methods:**

We retrospectively analyzed adults with refractory cardiac failure after open-heart surgery who required a venoarterial (VA) ECMO between 2016 and 2021.

**Results:**

Of 16 patients with VA ECMO, 60% were men (n=10), 90% had hypertension (n=14), 69% had < 30% of left ventricular ejection fraction (n=11), and the mean European System for Cardiac Operative Risk Evaluation II score was 12 ± 11%. ECMO support with central cannulation accounts for 81% (n=13), and an intra-aortic balloon pump was used in nine patients (56%). The mean time of support was 4.7 ± 2.6 days (1.5 - 12 days). ECMO weaning was achieved in 88% of patients, and in-hospital mortality was 44% (n=7) after discharge. The freedom from all-cause mortality at one year of follow-up of the entire cohort was 38% (n=6).

**Conclusion:**

VA ECMO is now a well-known life-saving therapeutic option, but mortality and morbidity remain high. Implementation of an ECMO program with educational training is mandatory in order to find the proper balance between patient benefits, ethical considerations, and public health financial input in South America.

## INTRODUCTION

**Table:** 

Abbreviations, Acronyms & Symbols				
AMI	= Acute myocardial infarction		IABP	= Intra-aortic balloon pump
BMI	= Body mass index		ICU	= Intensive care unit
BSA	= Body surface area		LVEF	= Left ventricular ejection fraction
CABG	= Coronary artery bypass grafting		MAP	= Mean arterial pression
CPB	= Cardiopulmonary bypass		OR	= Operating room
DM	= Diabetes mellitus		SD	= Standard deviation
ECMO	= Extracorporeal membrane oxygenation		SvO₂	= Venous oxygen saturation
EuroSCORE	= European System for Cardiac Operative Risk Evaluation		VA	= Venoarterial
HBP	= High blood pressure			

The incidence of cardiogenic shock following open-heart surgery has been reported between 3% and 5%^[1]^. Despite most of the patients could be weaned from cardiopulmonary bypass (CPB) with pharmacotherapy and intra-aortic balloon pump (IABP), at least 1% evolves with progressive organ dysfunction in spite of optimized management and needed advanced mechanical circulatory support^[2]^. Extracorporeal membrane oxygenation (ECMO) is the first line mechanical circulatory support, which allows time for “bridge to decision” or “bridge to recovery” for rescue patients in refractory cardiocirculatory failure^[3,4]^. ECMO is a clinically demanding procedure, in which a multidisciplinary approach is required, with significant financial burden to the institution and public health system. The latter has hampered their use in Latin America.

The objective of this study was to assess the impact of ECMO in patients with cardiogenic shock after cardiac surgery in a single center with ECMO unit in Chile.

## METHODS

### Study Population

Baseline characteristics, perioperative data, and in-hospital outcomes were prospectively collected in the computerized cardiac surgical database of Las Higueras Hospital (Talcahuano, Chile). We retrospectively analyzed all adults with refractory cardiac failure after open-heart surgery who required an ECMO implantation between 2016 and 2021. During this period, 1947 patients underwent cardiac surgery, and 16 patients developed a post cardiotomy shock requiring mechanical support. Patients who required ECMO support as first intention (cardiogenic shock in non-surgical patients) as well as those with lung disease, such as hantavirus pulmonary syndrome or adult respiratory distress syndrome, were excluded.

The study protocol was approved by Las Higueras Hospital ethics committee, and the need for informed consent was waived by the board. For this publication, the instructions for authors and ethical responsibilities have been taken into account. All research was conducted in accordance with relevant guidelines/regulations. This project was approved by the Scientific Ethics Committee of the Ministry of Health (Act No. 102 of 11.09.2021).

### ECMO Use Strategy and Indications

ECMO was indicated in patients undergoing cardiac surgery when the maximal use of pharmacological agents (two inotropic or vasopressor agents) and the IABP was not enough to wean off CPB or when the low cardiac output is persistent in the intensive care unit (ICU) despite optimized management, prior to severe end-organ hypoperfusion. The contraindications in our group were an uncontrollable bleeding and severe neurological damage. We preferred not to support patients older than 70 years, because advanced age has been associated with worse outcomes. The indications for ECMO were supported by transesophageal echocardiogram or a Swan Ganz catheter with a poor univentricular or biventricular function, and persistent low cardiac output in patients with data suggesting the possibility of improvement after ventricular assistance. We considered ECMO as a “bridge to recovery” in all patients and as a “bridge to heart transplant” in those younger than 65 years old. We stated that ECMO was not used as a bridge for transplantation in this cohort.

### Patient Management Strategy

The venoarterial (VA) ECMO flow was programmed according to the patient’s body surface area (BSA) in order to deliver 2.4 L/min/m^2^, and it was adjusted according to the patient’s hemodynamic requirements and oxygen demand. Monitored variables were lactic acid, mixed venous oxygen saturation (SvO₂) between 65% and 70%, oxygen blood pressure > 60 mmHg, and carbon dioxide in the range of 35-45 mmHg. During VA ECMO support, anticoagulation with heparin was started in patients without active bleeding signs (drain balance < 200 cc/hour). When anticoagulation was indicated, the initial dose was of 10 U/kg/hour with a target of activated partial thromboplastin time of 50-65 seconds, monitored every six hours. Protective mechanical ventilation was maintained during ECMO support.

To maintain an optimum hemodynamic profile (mean arterial pressure [MAP] 65-70 mmHg), inotropic agents (vasopressin, noradrenaline, and adrenaline) were titrated to optimize the myocardial function, always promoting aortic valve opening and maintaining a > 15 mmHg differential pression during the initial stages. An IABP was used in patients with refractory cardiogenic shock after coronary artery bypass grafting (CABG) in order to reduce the afterload, and to increase the coronary perfusion and pulsatility. Once connected to VA ECMO, the IABP was maintained as a strategy for left ventricular decompression.

ECMO weaning criteria were: MAP > 65 mmHg; use of one vasopressor or two at low doses; pulse pressure > 20 mmHg; central venous pressure < 8-10 mmHg; SvO₂ > 65%; left ventricular ejection fraction (LVEF) > 25% and velocity time integral > 12 cm; normal chest X-ray; and absence of multi-organ dysfunction. Once patients fulfilled these conditions, the flow was reduced by 25% every eight hours until the patient reached a two liters flow. Afterwards, the patients were moved to the operating room (OR) for withdrawing the cannulas, assisted by a transesophageal echocardiogram and with very close hemodynamics surveillance.

The VA ECMO perfusion system consists of a centrifugal pump (Rotaflow and Medos), a polymethylpentene oxygenator (Maquet PLS and Medos Hilite 7000), and a heat exchanger (Hico-Aquatherm 660). The arterial return cannula (19 F to 21 F) was inserted directly to the ascending aorta (n=13) and to the femoral artery (n=3). Venous return was obtained with a two-phase 29 F to 32 F cannula inserted directly into the right atrium (n=5); and, in patients with mixed and peripheral cannulation, cannulas of 23 Fr and 25 F were used in the femoral vein (n=8) with the tip inserted in the cavoatrial junction (n=3).

### Variables for Analysis

The following variables were analyzed for each patient: i) preoperative variables: type of surgical procedure, age (years), gender, weight (kg), height (cm), body mass index (BMI) (kg/cm^2^), BSA (m^2^), European System for Cardiac Operative Risk Evaluation (EuroSCORE) II, and LVEF (%) - hypertension, diabetes mellitus, acute myocardial infarction, cardiopulmonary arrest, and type of surgery (elective, urgent, emergency, and salvage) were defined according to the EuroSCORE II definition^[5]^ -; ii) intraoperative variables: type of cannulation (central and peripheral); CPB time (min), aortic cross-clamping time (min), drug use (adrenalin, noradrenaline, dobutamine, and milrinone), and use of IABP; iii) support: days of support; iv) laboratory: lactic acid (mg/dL).

### Statistical Analysis

The distribution of baseline variables and perioperative events are reported as mean ± standard deviation or median with interquartile range for continuous variables and proportions for categorical variables. Nonparametric estimates of freedom from all-cause mortality were performed using a Kaplan-Meier model. A Kaplan-Maier curve with lactic acid 24 hours after an ECMO implantation and mortality was made categorizing this last as < 10 mg/dL or > 10 mg/ dL. Statistical significance was present when *P*-value was < 0.05. Analyses were performed using IBM Corp. Released 2019, IBM SPSS Statistics for Windows, version 26.0, Armonk, NY: IBM Corp.

### ECMO Protocol

As a well-established consensus or guidelines do not exist about ECMO utilization in those patients, its use is left to every institution’s protocol and experience. We have a consensus on ECMO support in postcardiotomy^[6]^ already published, and a reference should be included in your statement if you disagree.

## RESULTS

Patient’s characteristics are shown in Table 1; 60% of them were men (n=10), 90% had hypertension (n=14), and 69% had left ventricular disfunction (n=11, X=25 ± 17). The mean EuroSCORE II was 12 ± 11, and Survival after Veno-Arterial ECMO score was -3; most of surgeries were performed in an urgent or emergency status. As several series, ECMO was associated with CABG in n=10 patients (62%), and six patients (38%) with complex cardiac surgery. Aortic cross-clamping time and CPB time are shown in Table 2.

**Table 1:** Baseline characteristics of the entire cohort.

Preoperative variables	Total n=16 patients (%) min - max
Gender	
Male	10 (60%)
Female	6 (40%)
Age, years (mean ± SD)	58 (± 8.2)
Comorbidity	
HBP	14 (87.5%)
Recent AMI	9 (56.3%)
BMI	29 (± 5.4)
DM	5 (31.3%)
EuroSCORE II	12 (± 11) (1.3-39)
Left ventricular function	25 (± 17) (10-59)
Indication	
Salvage	2 (12%)
Emergency	3 (19%)
Urgent	11 (69%)

AMI=acute myocardial infarction; BMI=body mass index; DM=diabetes mellitus; EuroSCORE=European System for Cardiac Operative Risk Evaluation; HBP=high blood pressure; SD=standard deviation

**Table 2:** Intraoperative characteristics of the whole cohort.

Intraoperative variables	Total n=16 patients (%) min - max
CPB time (mean ± SD)	150 (± 87) (58-327)
Aortic cross-clamping time (mean ± SD)	96 (± 62) (32-247)
Vasoactive drugs utilization	Noradrenaline 14 (88%)
	Adrenaline 8 (50%)
	Milrinone 4 (25%)
	Dobutamine 3 (19%)
Previous cardiac surgery	3 (19%)
Isolated CABG	10 (62%)
Complex cardiac procedures	6 (38%)

CABG=coronary artery bypass grafting; CPB=cardiopulmonary bypass; SD=standard deviation

ECMO is most often used for failure to wean from CPB, hence, we preferred to use the central or the mix cannulation in the OR (80%, n=13). Three (20%) patients were connected by peripheral cannulations using a cutdown technique in the ICU. The duration of support was 4.7 ± 2.6 days (1.5 - 12 days), which reflected that the recovery was earlier after cardiac surgery, and the weaning process was achieved in 88% (n=14). Although VA ECMO is the first line support in this pathology, IABP was used as the first approach in 56% of patients (n=9) undergoing CABG. We know that the benefit of the concomitant use of IABP with ECMO is unclear, however, once the IABP was implanted and the ECMO support was required, we preferred a simultaneous support aiming for left ventricular decompression and enhanced left side performance.

**Fig. 1:**
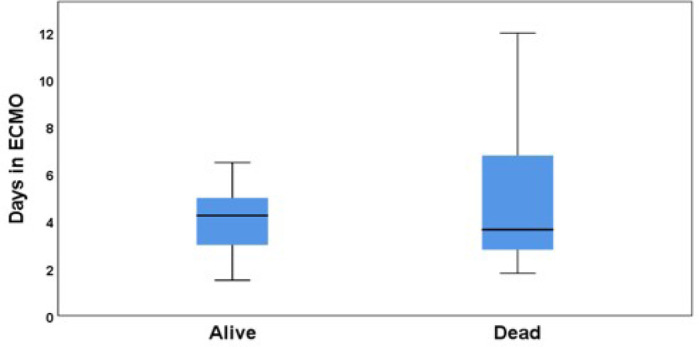
Blog-spot analysis of the time (days) in extracorporeal membrane oxygenation (ECMO) assistance and its relationship with mortality.

**Fig. 2:**
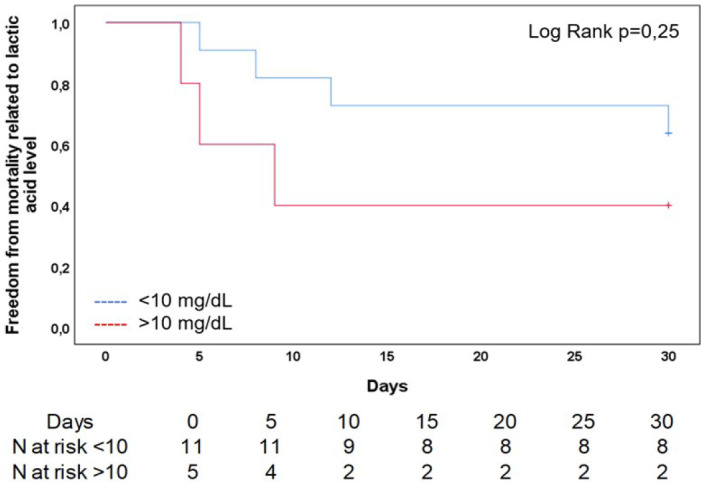
Freedom from mortality at 30 days for the entire cohort of patients and its relationship with lactic acid level 24 hours after extracorporeal membrane oxygenation assistance.

**Fig. 3:**
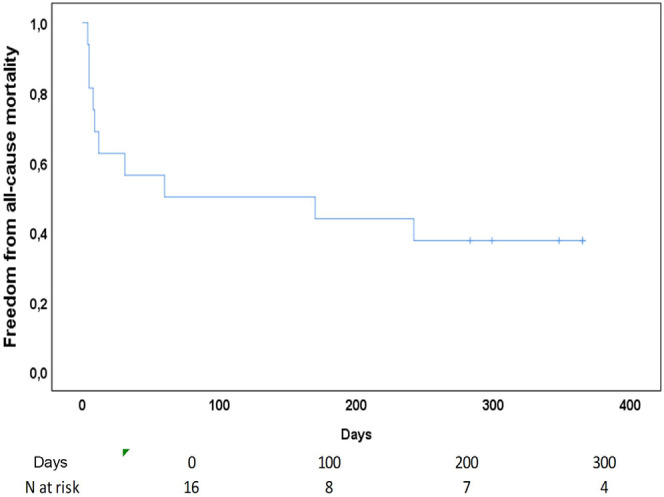
Kaplan-Meier curve showing a freedom from all-cause mortality of the entire cohort at one year of follow-up.

Complications in ECMO are common and increase over the time (Table 3); as it is shown in (Figure 1), patients who required longer ECMO support had higher mortality. The most frequent complication was surgical bleeding, requiring reoperation by cardiac tamponade in 56% of patients (n=9). Limb ischemia occurred in seven patients, two with peripheral cannulation and five with central cannulation. As it was expected, a higher mortality was highlighted in those with higher lactic acid level after 24 hours of ECMO assistance (Figure 2). In-hospital mortality was 44% (n=7) after discharge, three patients died, all of them by cardiac causes at follow-up. The freedom from all-cause mortality at one year of follow-up of the entire cohort was 38% (n=6) (Figure 3).

**Table 3:** Complications during extracorporeal membrane oxygenation.

Type of complications	Total n=16 patients (%)
Surgical site bleeding	13 (81%)
Neurologic events	
Encephalopathy	1 (6%)
Stroke	2 (12%)
Sepsis	
Pneumonia	1 (6%)
Deep sternal wound infection	1 (6%)
Bacteremia	3 (19%)
Limb complications	
Ischemia	5 (31%)
Amputation	2 (12%)
Cardiac tamponade	9 (56%)
Type B aortic dissection	2 (12%)

## DISCUSSION

Our ECMO program was started in 2016 to treat three prevalent pathologies in our country: i) hantavirus pulmonary syndrome, ii) adult respiratory distress syndrome, and iii) cardiogenic shock. This study aims to show our initial experience with VA ECMO utilization in patients with refractory cardiogenic shock after cardiac surgery in one single institution in Chile.

The incidence of VA ECMO utilization after open-heart surgery has been reported between 0.4 and 3.7%^[7]^. Accordingly, we reported an incidence of 0.82% of ECMO utilization after open-heart surgery in patients with refractory cardiogenic shock. Although our reported incidence was low, we had a higher survival rate (38%) after one year of intervention, compared to the studies by Wang et al^[12]^. (34%) and Khorsandi et al.^[10]^ (31%)^[8]^. We could explain these differences due to the baseline characteristics of the patients included in other reports, such as, older patients, with prior myocardial infarction, usually had left main coronary disease, left ventricular dysfunction, prior open-heart surgery, and were frequently associated with combined surgeries^[9]^.

Interestingly, our data showed similar characteristics to almost 70% of the patients with severe left ventricular dysfunction, such as: i) > 60% of CABG in non-elective patients, and ii) high-risk scores (mean EuroSCORE II 12). Conversely, by the fact that the results showed in this article were the first data with ECMO utilization after cardiac surgery, which included our learning curve as team, our age population was younger than previous reports (58 years old).

In this cohort, the implementation of ECMO in all patients was unplanned, and the decision for support was guided by an increased requirement for vasoactive drugs followed by a close evaluation by transesophageal echocardiography in order to establish a true refractory cardiogenic shock. After that period, considering the high prevalence of patients with coronary disease, an IABP was utilized according to our institutional protocol, which explains that 56% of patients received one previously to ECMO implantation.

Despite the presence of an open-heart surgery, peripheral cannulation was frequently used as it reduces the possibility of mediastinal infection, avoids resternotomy, and allows for an uninterrupted chest compression during ECMO cannulation^[6,7]^. Our institutional cannulation strategy was central cannulation if the patient’s sternum was still opened in the OR, that’s why only three patients had peripheral cannulation installed in the ICU unit.

In the other hand, central cannulation was associated with higher rates of bleeding but no difference in overall survival when compared with peripheral cannulation^[7,11]^. We reported bleeding as the most frequent complication (81%) in our cohort because of recent surgery, usually with fragile tissue due to a reoperation and the early need of anticoagulation.

The mean duration of ECMO support was 4.7 days, as other publications who advocate that 48 to 72 hours support times are enough time to start the weaning. If insufficient recovery was observed and a more advanced mechanical support or a heart transplantation was needed, we transferred them to a center that perform heart transplantation^[12]^.

As it was reported by others^[7]^, we also founded a higher mortality but it was not statistically significant in patients with > 10 mg/dL lactic acid level after 24 hours of ECMO assistance. These patients were in salvage status or had a BMI > 34.

In spite of some enthusiastic ranging of weaning from VA ECMO (31-76%)^[7]^, we found that this was not correlated with the dramatic in-hospital mortality. We have had achieved 14 weaning in these five years, but our in-hospital mortality was 44%, and the principal causes were sepsis and arrhythmias. Doll et al.^[13]^ reported 76% in-hospital mortality over 219 patients after post cardiotomy refractory shock. Wang et al.^[12]^, in a meta-analysis of 20 observational studies, founded 34% of in-hospital mortality and one-year survival rate of 24%. Other metanalysis of Khorsandi et al.^[10]^ with 24 retrospective studies shows survival to in-hospital discharge of 30.8%.

### Limitations

Limitations of this article are related to its retrospective nature, the bias of patient’s selection when an ECMO program is started, and those related to patients’ data collection.

## CONCLUSION

Patients with refractory cardiogenic shock after cardiac surgery are still a very vulnerable population with a life-threatening condition. VA ECMO is now a well-known life-saving therapeutic option, but mortality and morbidity remain high. Our results are not by chance, managing these patients requires an ECMO program with a multidisciplinary teamwork with continuous training aiming to find the proper balance between patients benefits, ethical considerations, and public health financial input in South America.
